# Clinical and Epidemiological Characteristics of Mortality in Patients With Polytrauma

**DOI:** 10.7759/cureus.114307

**Published:** 2026-08-10

**Authors:** Cristian P Castro

**Affiliations:** 1 Surgery, Universidad de San Carlos de Guatemala, Guatemala City, GTM

**Keywords:** accidents, emergencies, mortality, multiple trauma, wounds and injuries

## Abstract

Introduction

Polytrauma constitutes one of the leading causes of morbidity and mortality worldwide, representing a significant public health problem that primarily affects the working-age population. It is defined as the simultaneous presence of multiple traumatic injuries affecting different organs and systems, resulting in a critical condition that requires a rapid, coordinated, and multidisciplinary response.

Methods

A retrospective, descriptive study was conducted among 229 patients who died following polytrauma at Coatepeque National Hospital between January 2020 and January 2025. The study included only patients with a documented in-hospital outcome. Therefore, patients transferred to a higher-level hospital, those discharged alive, and those discharged against medical advice were excluded. The study variables included the epidemiological profile (age, sex, and comorbidities) and characteristics of the injury (mechanism of trauma and primary cause of death). The mechanism of trauma was classified as blunt or penetrating, whereas the primary cause of death was classified into the following categories: traumatic brain injury, cervical trauma, thoracic trauma, abdominal trauma, pelvic trauma, extremity trauma, and other causes. The “other” category included spinal cord trauma, burns, and crush injuries.

Results

The study identified an in-hospital mortality rate of 6%, corresponding to 229 deaths. Among the patients who died, 175 (76.4%) were male and 54 (23.6%) were female. The 21-30-year age group represented the largest proportion of deaths, with 76 cases (33.2%), followed by the 31-40-year age group with 35 cases (15.3%) and the 71-80-year age group with 24 cases (10.5%). Traumatic brain injury was the most frequently documented cause of death, accounting for 160 cases (69.9%), followed by thoracic trauma with 24 cases (10.5%), abdominal trauma with 18 cases (7.9%), extremity trauma with 12 cases (5.2%), pelvic trauma with 10 cases (4.4%), cervical trauma with three cases (1.3%), and other causes with two cases (0.9%). Regarding the mechanism of injury, blunt trauma accounted for 173 cases (75.5%), whereas penetrating trauma accounted for 56 cases (24.5%). Most patients had no documented comorbidities, representing 180 cases (78.6%); among patients with documented comorbidities, diabetes mellitus was present in 23 cases (10.0%) and hypertension in 12 cases (5.2%).

Conclusion

The study provides baseline clinical and epidemiological information on in-hospital polytrauma mortality at the institution, contributing to the characterization of the affected population and establishing an institutional evidence base that may support clinical decision-making, healthcare planning, and future analytical research.

## Introduction

Polytrauma

Polytrauma is defined as the simultaneous presence of two or more traumatic injuries in the same individual, with at least one posing a potential threat to life. According to the Berlin definition, polytrauma is characterized by injuries with an Abbreviated Injury Scale (AIS) score of ≥3 in at least two body regions, together with at least one of the following criteria: age ≥70 years, hypotension, impaired level of consciousness, acidosis, or coagulopathy [1].

Worldwide, deaths and injuries resulting from road traffic accidents continue to represent a major public health problem. Approximately 1.19 million deaths and between 20 and 50 million nonfatal traumatic injuries are recorded each year as a result of these events. The burden of this problem is not distributed evenly, as low- and middle-income countries bear a disproportionately greater share of its consequences [2].

Pathophysiology

Trauma is a condition that affects the entire body. The initial response to trauma triggers a complex neuroendocrine reaction that significantly affects cardiac function, intravascular volume, and metabolism. Subsequently, an inflammatory response develops, which is associated with alterations in the endothelium and in the regulation of blood volume, body temperature, coagulation, and organ function. The devastating effects of severe trauma are directly related to blood loss and the resulting hypoperfusion. The most significant and dangerous consequences are collectively known as the “lethal triad,” which includes hypothermia, acidosis, and coagulopathy. This concept was initially described by researchers in Denver [3].

Types of trauma

Traumatic Brain Injury

Traumatic brain injury may result from various external physical forces acting on the head. It may involve a combination of primary and secondary injuries. The primary injury, which occurs immediately after the impact, may be caused by the sudden displacement of the brain due to a direct impact, sudden acceleration or deceleration, or the penetration of a foreign object. Subsequently, a secondary neurotoxic response may be activated, leading to cerebral edema, cerebral hypoxia, increased levels of neurotoxic substances, the release of free radicals, and increased intracranial pressure [4].

One of the most serious injuries associated with traumatic brain injury is intracranial hemorrhage, a condition that encompasses several types of bleeding within the skull, including epidural hemorrhage, subdural hemorrhage, subarachnoid hemorrhage, and intraparenchymal hemorrhage [5].

Cervical Trauma

Several vital organs and structures are located in close proximity within this anatomical region. Therefore, injuries that appear minor may result in severe damage or potentially fatal complications, either immediately or at a later stage. For descriptive and clinical management purposes, the neck is divided into three zones: 1, 2, and 3. Zone 1 contains important structures such as the innominate vessels, the origin of the common carotid artery, the subclavian arteries, the vertebral artery, the brachial plexus, the trachea, the esophagus, the lung apex, and the thoracic duct. Zone 2 is the largest region and the one most frequently affected by injuries. Among the structures it contains are the carotid and vertebral arteries, the internal jugular veins, the trachea, and the esophagus. Zone 3 contains the distal portions of the carotid and vertebral arteries, along with the pharynx. Due to its proximity to the base of the skull, this region is less accessible to physical examination and represents a challenge during surgical evaluation [6].

Thoracic Trauma

Thoracic trauma represents a considerable challenge, as it encompasses a wide range of injuries, from minor rib fractures to severe intrathoracic organ injuries that pose a threat to life. Accidents, falls, and motor vehicle collisions are common causes of thoracic injuries. Injuries to vital organs located within the thorax may progress rapidly. Both blunt and penetrating thoracic trauma can result in various complications, including tension pneumothorax, open pneumothorax, hemothorax, flail chest, pericardial effusion, cardiac tamponade, aortic rupture, esophageal injury, tracheobronchial tree injury, cardiac or pulmonary contusion, and diaphragmatic rupture [7].

Abdominal Trauma

Due to its impact on morbidity and mortality, abdominal trauma requires prompt evaluation and management. It is classified as blunt or penetrating trauma. Blunt abdominal trauma results from blunt forces applied to the abdominal wall without disruption of its integrity, primarily affecting solid organs such as the liver, spleen, and kidneys because of their rich vascular supply. One of its main challenges lies in its nonspecific clinical presentation and the possibility that some injuries may go unrecognized during the initial evaluation [8]. In contrast, penetrating abdominal trauma occurs when a foreign object passes through body tissues and is commonly caused by firearms, stab wounds, explosions, or high-velocity impacts. The severity of these injuries depends on factors such as the amount of energy transferred and the trajectory of the object [9].

*Pelvic* *Trauma*

The pelvis is a strong bony structure that requires a considerable amount of energy to sustain a fracture. When a pelvic fracture or ligamentous injury occurs, the volume of the pelvic cavity may increase, reducing spontaneous tamponade and promoting the accumulation of blood within the retroperitoneal space. Approximately 25% of patients with severe trauma present with pelvic fractures, which are associated with a high mortality rate, primarily due to massive hemorrhage and concomitant injuries outside the pelvis [10].

Diagnostic resources

Imaging techniques are fundamental to the initial evaluation of patients with polytrauma. Conventional radiography remains important for detecting injuries involving the thorax, cervical spine, and pelvis. FAST and EFAST USG allow for the rapid identification of hemorrhage within the abdominal, pericardial, and thoracic cavities, whereas CT is an indispensable tool for diagnosing severe trauma due to its speed and accuracy in detecting injuries. MRI is not routinely used in emergency situations and is primarily reserved for evaluating spinal cord injuries or injuries involving the pancreatic duct [11].

Mortality in polytrauma

Mortality in polytrauma follows a trimodal distribution consisting of immediate, early, and late mortality. Immediate mortality occurs at the scene of the incident or shortly thereafter and is typically associated with severe brain or cardiovascular injuries. Early mortality occurs within the first 24 hours after trauma and is generally related to potentially reversible conditions, whereas late mortality occurs days after the injury and is primarily associated with sepsis, coagulation disorders, and multiple organ failure [12].

Despite advances in trauma systems and multidisciplinary management, polytrauma continues to be associated with substantial in-hospital mortality worldwide. Mortality patterns vary according to demographic characteristics, mechanisms of injury, injury severity, available healthcare resources, and institutional capabilities. Therefore, generating institution-specific clinical and epidemiological evidence remains essential for characterizing local mortality patterns, identifying the most frequent causes of death, and providing information that may support healthcare planning and future research.

## Materials and methods

A descriptive, cross-sectional, and retrospective study was conducted through a review of 229 medical records of patients who died following polytrauma at the National Hospital of Coatepeque between January 2020 and January 2025. During the same period, a total of 3,820 patients were hospitalized for polytrauma, and this population was used exclusively as the denominator to estimate in-hospital mortality. The descriptive analysis was limited to patients who died during hospitalization. Patients transferred to higher-level hospitals, patients discharged alive, and those who left against medical advice were excluded because the study was designed to characterize in-hospital mortality and the clinical and epidemiological profile of deceased patients.

The study variables included the epidemiological profile (age, sex, and comorbidities) and incident-related characteristics (mechanism of trauma and primary cause of death). The mechanism of trauma was classified as blunt or penetrating, whereas the primary cause of death was classified into the following categories: traumatic brain injury, cervical trauma, thoracic trauma, abdominal trauma, pelvic trauma, extremity trauma, and other causes. The “other causes” category included spinal cord trauma, burns, and crush injuries. Data were collected using a standardized data collection form, which is provided in Appendix 1.

A univariate descriptive statistical analysis was performed, and each variable was summarized using absolute and relative frequencies. In-hospital mortality was calculated as the proportion of patients who died from polytrauma relative to the total number of patients hospitalized for this condition during the study period. In accordance with the descriptive design of the study, no comparative analyses or inferential statistical tests were performed.

The study was conducted in accordance with ethical principles, ensuring the confidentiality and anonymity of the information contained in the medical records. Due to the retrospective nature of the study and the exclusive use of anonymized data, no intervention involving patients was performed. The information obtained was used solely for academic and scientific purposes.

## Results

Between January 2020 and January 2025, a total of 3,820 patients were hospitalized for polytrauma at Hospital Nacional de Coatepeque “Juan José Ortega.” Of these, 229 patients died, corresponding to an in-hospital mortality rate of 6%. The in-hospital mortality indicators are presented in Table 1.

**Table 1:** In-hospital mortality among patients with polytrauma.

Indicator	Result
Total patients hospitalized for polytrauma	3,820
In-hospital deaths	229
In-hospital mortality rate	6.00%

Of the 229 patients who died, 175 (76.4%) were male and 54 (23.6%) were female. The age distribution included 76 cases (33.2%) in the 21-30-year age group, 35 cases (15.3%) in the 31-40-year age group, and 24 cases (10.5%) in the 71-80-year age group. No documented comorbidities were recorded in 180 cases (78.6%). Among the documented comorbidities, diabetes mellitus accounted for 23 cases (10.0%) and hypertension for 12 cases (5.2%). The remaining comorbidities were recorded less frequently. The epidemiological profiles and comorbidities of the deceased patients are presented in Table 2.

**Table 2:** Epidemiological profile and comorbidities of patients who died following polytrauma (n = 229).

Variable	Category	Frequency (n)	Percentage (%)
Sex	Male	175	76.4
	Female	54	23.6
Age (years)	1-10	4	1.7
	11-20	23	10
	21-30	76	33.2
	31-40	35	15.3
	41-50	20	8.7
	51-60	20	8.7
	61-70	18	7.9
	71-80	24	10.5
	81-90	6	2.6
	91-100	3	1.3
Comorbidity	Hypertension	12	5.2
	Diabetes mellitus	23	10
	Heart disease	2	0.9
	Obesity	4	1.7
	Other comorbidities	8	3.5
	None	180	78.6

Among the mechanisms of trauma, blunt trauma was documented in 173 cases (75.5%) and penetrating trauma in 56 cases (24.5%). The distribution of the primary causes of death was as follows: traumatic brain injury in 160 cases (69.9%), thoracic trauma in 24 cases (10.5%), abdominal trauma in 18 cases (7.9%), extremity trauma in 12 cases (5.2%), pelvic trauma in 10 cases (4.4%), cervical trauma in three cases (1.3%), and other traumatic causes in two cases (0.9%). The distributions of the mechanisms of trauma and primary causes of death are presented in Table 3.

**Table 3:** Mechanisms of trauma and primary causes of death among patients who died following polytrauma (n = 229).

Variable	Category	Frequency (n)	Percentage (%)
Mechanism of trauma	Blunt trauma	173	75.5
	Penetrating trauma	56	24.5
Primary cause of death	Traumatic brain injury	160	69.9
	Cervical trauma	3	1.3
	Thoracic trauma	24	10.5
	Abdominal trauma	18	7.9
	Pelvic trauma	10	4.4
	Extremity trauma	12	5.2
	Other traumatic causes	2	0.9

## Discussion

During the study period, 3,820 patients were hospitalized for polytrauma, of whom 229 died, corresponding to an in-hospital mortality rate of 6.0%. This mortality rate was lower than the 10.2% reported in the Spanish National Polytrauma Registry by Campos-Serra A et al. in a cohort of 2,069 patients treated at high-complexity hospitals in Spain [13]. Differences between these findings should be interpreted in light of the characteristics of the study populations and the organization of trauma care. At Hospital Nacional de Coatepeque “Juan José Ortega,” patients requiring highly specialized procedures, such as neurosurgery or cardiothoracic surgery, are routinely referred to higher-level healthcare facilities, whereas the present study evaluated only the in-hospital outcomes documented at the hospital. In another clinical setting, Cabrero HM et al. reported a mortality rate of 14.3% among pediatric patients with severe traumatic brain injury [14]. This finding further highlights the differences between the study populations included in each investigation.

Compared with the previously cited studies reporting higher mortality rates among patients with polytrauma, the in-hospital mortality observed in the present study was lower. This difference should be interpreted in the context of the study design and the established exclusion criteria. Patients discharged against medical advice and those referred to higher-level healthcare facilities were excluded because their subsequent clinical outcomes could not be verified through the institutional records. Consequently, the mortality estimate presented in this study reflects only the documented in-hospital outcomes of the study population. The absence of follow-up data for excluded patients represents an important limitation and may have contributed to an underestimation of mortality among patients initially treated at the institution.

Among the patients who died, 175 (76.4%) were male and 54 (23.6%) were female. A similar distribution was reported in the Spanish National Polytrauma Registry, where Cabrero HM et al. found that more than three-quarters of patients with polytrauma were men [13]. Likewise, Macedo Camargos de Oliveira CH et al. reported that 63.3% of trauma patients transported by air were male. Although the study populations and clinical settings differed, these findings indicate a consistent predominance of male patients among individuals with traumatic injuries across the cited studies [15-16].

Regarding age distribution, 76 cases (33.2%) corresponded to the 21-30-year age group, followed by 35 cases (15.3%) in the 31-40-year age group. This distribution is consistent with findings reported at Pedro de Bethancourt Hospital in Antigua Guatemala, where Ixcoy Tocay and Durán Campos documented that 68% of patients involved in motorcycle accidents were between 18 and 29 years of age and reported a high frequency of helmet nonuse [16]. Similarly, Campos-Serra A et al. reported a mean age of 45 years among patients with polytrauma in the Spanish National Polytrauma Registry [13]. In addition, 24 cases (10.5%) corresponded to patients aged 71-80 years. Although the study populations and clinical settings differed, the cited studies reported similar age distributions among patients with traumatic injuries. These findings underscore the importance of considering age distribution when planning trauma prevention and healthcare strategies.

Regarding the mechanism of injury, 173 cases (75.5%) corresponded to blunt trauma, compared with 56 cases (24.5%) of penetrating trauma. This distribution is consistent with findings from the Spanish National Polytrauma Registry, where Campos-Serra A et al. reported that 80% of cases involved blunt trauma, with motorcycle accidents being the most frequent mechanism of injury, whereas penetrating trauma accounted for 12% of cases, mainly due to stab wounds [13]. Similarly, Arbizu FE et al. reported a predominance of blunt trauma and found that head and thoracic injuries were associated with mortality [17]. These findings are consistent with our results regarding the distribution of trauma mechanisms. Although penetrating trauma was less frequent in our series, it remains an important cause of traumatic mortality because of the severity of the injuries it may produce. This highlights the need for rapid care pathways, hemorrhage-control protocols, emergency surgical intervention, and the timely availability of surgical resources.

Traumatic brain injury was the most frequently documented cause of death, accounting for 160 cases (69.9%), followed by thoracic trauma with 24 cases (10.5%), abdominal trauma with 18 cases (7.9%), extremity trauma with 12 cases (5.2%), pelvic trauma with 10 cases (4.4%), cervical trauma with three cases (1.3%), and other traumatic causes, including burns, spinal cord trauma, and crush injuries, collectively accounting for two cases (0.9%). This distribution is consistent with the literature, as Arbizu FE et al. reported that head and thoracic injuries were among the most frequently affected anatomical regions and were associated with mortality among patients with severe trauma [17]. Likewise, Cabrero HM et al. reported a mortality rate of 14.3% among pediatric patients with severe traumatic brain injury, highlighting the high mortality associated with severe neurotrauma [14]. At the national level, Ixcoy Tocay and Durán Campos described moderate and severe traumatic brain injury resulting from motorcycle accidents among adults treated at Pedro de Bethancourt Hospital in Antigua Guatemala [16]. These findings are consistent with the literature, which identifies traumatic brain injury as one of the principal causes of death among patients with polytrauma. Furthermore, factors such as limited access to advanced imaging studies, the restricted availability of neurosurgical services in regional hospitals, and delays in referral to specialized centers may partially contribute to the high proportion of deaths attributed to traumatic brain injury in this population.

Regarding comorbidities, 180 patients (78.6%) had no documented comorbidities. Among those with chronic diseases, the most frequent comorbidities were diabetes mellitus in 23 cases (10.0%), hypertension in 12 cases (5.2%), obesity in four cases (1.7%), and cardiovascular disease in two cases (0.9%). Other comorbidities, including chronic liver disease, active cancer, asthma, and chronic kidney disease, collectively accounted for eight cases (3.5%). These findings indicate that, although most deceased patients with polytrauma had no documented comorbidities, chronic medical conditions were present in 49 cases (21.4%). Burton KR and Magidson PD noted that older adults have a higher prevalence of comorbidities and reduced physiological reserve, increasing their vulnerability to trauma [18]. Although our study was not designed to establish causal associations, the findings are consistent with the literature, which recognizes comorbidities as an important consideration in the clinical management of patients with traumatic injuries.

## Conclusions

The in-hospital mortality rate among patients admitted with polytrauma at Hospital Nacional de Coatepeque during the study period was 6.0%. Among the deceased patients, the highest proportions were observed among males and patients in the 21-30-year age group. Traumatic brain injury was the most frequently documented cause of death, whereas blunt trauma was the most frequently documented mechanism of injury. Most deceased patients had no documented comorbidities, while diabetes mellitus and hypertension were the most frequent chronic conditions among those with pre-existing diseases. Together, these findings provide an epidemiological characterization of in-hospital mortality among patients with polytrauma treated at the institution and contribute to a better understanding of the demographic and clinical profile of this population. The results may support the planning of trauma-prevention initiatives and the optimization of trauma care, particularly regarding the timely recognition and management of traumatic brain injury.

## Figures and Tables

**Figure 1:**
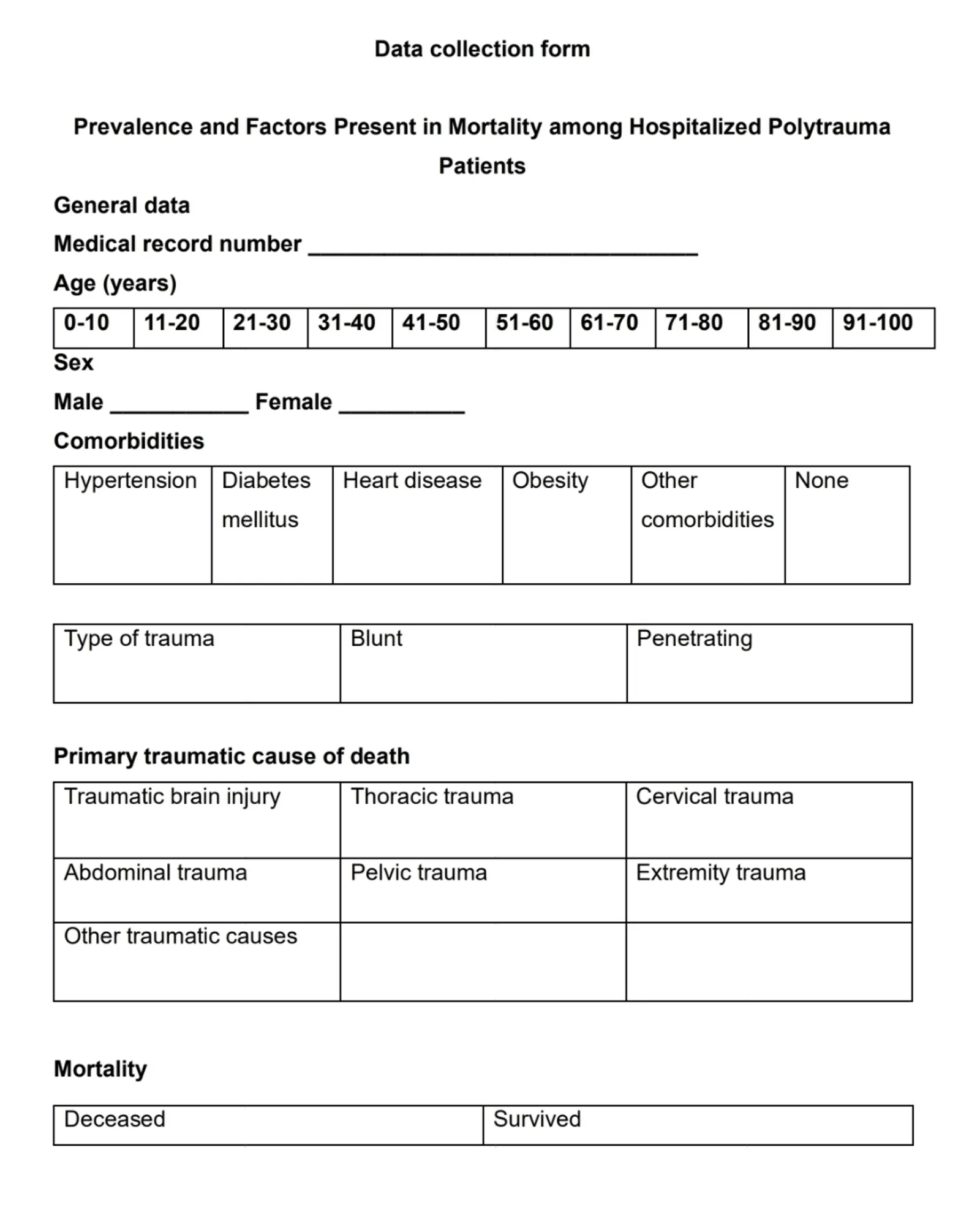
Data collection form.

## Appendices

Appendix 1

## References

[1] Upadhyaya GK, Iyengar KP, Jain VK, Garg R (2021). Evolving concepts and strategies in the management of polytrauma patients. J Clin Orthop Trauma.

[2] (2026). Global status report on road safety 2023 [Internet]. Geneva: World Health Organization. https://www.who.int/publications/i/item/9789240086517.

[3] Pape HC, Moore EE, McKinley T, Sauaia A (2022). Pathophysiology in patients with polytrauma. Injury.

[4] Ginsburg J, Smith T (2026). Traumatic Brain Injury. https://www.ncbi.nlm.nih.gov/books/NBK557861/.

[5] Tenny S, Das JM, Thorell W (2026). Intracranial Hemorrhage Overview. https://www.ncbi.nlm.nih.gov/books/NBK470242/.

[6] Alao T, Waseem M (2026). Neck Trauma. https://www.ncbi.nlm.nih.gov/books/NBK470422/.

[7] Jain A, Waseem M (2026). Thoracic Trauma. https://www.ncbi.nlm.nih.gov/books/NBK482194/.

[8] Marrufo C, Mardely M (2023). Epidemiological characteristics and management of blunt abdominal trauma in patients treated in the surgery area of ​​the Cajamarca Regional Teaching Hospital between 2020-2022. https://alicia.concytec.gob.pe/vufind/Record/RUNC_c9d8cf238e7016d085f5710f1ff65883.

[9] Marietta M, Burns B (2026). Penetrating Abdominal Trauma. https://www.ncbi.nlm.nih.gov/books/NBK459123/.

[10] Mejia D, Parra MW, Ordoñez CA (2020). Hemodynamically unstable pelvic fracture: a damage control surgical algorithm that fits your reality. Colomb Med (Cali).

[11] Thippeswamy PB, Rajasekaran RB (2021). Imaging in polytrauma - principles and current concepts. J Clin Orthop Trauma.

[12] Espinoza JM (2011). Basic and advanced care of the polytrauma patient. Peruvian Med J.

[13] Campos-Serra A, Pérez-Díaz L, Rey-Valcárcel C (2023). Results of the Spanish National Polytrauma Registry. Where are we and where are we heading?. Cir Esp (Engl Ed).

[14] Cabrero HM, Iglesias Bouzas MI, Martínez de Azagra GA, Pérez SE, Serrano GA, Jiménez GR (2022). Early prognostic factors for morbidity and mortality in severe traumatic brain injury. Experience in a child polytrauma unit. Med Intensiva (Engl Ed).

[15] Kuhajda I, Zarogoulidis K, Kougioumtzi I (2014). Penetrating trauma. J Thorac Dis.

[16] Macedo Camargos de Oliveira CH, Gomes da Silva TR, Oliveira TM, de Carvalho FB, Corrêa dos AR (2021). Características dos atendimentos às vítimas de trauma admitidas em um pronto socorro via transporte aéreo. Rev Enferm Cent-Oeste Min.

[17] Arbizu FE, Galbete JA, Belzunegui OT, Fortún MM, Echarri SA (2024). Analysis of serious trauma injury patterns in Navarre (Spain) (2010-2019). An Sist Sanit Navar.

[18] Burton KR, Magidson PD (2023). Trauma (excluding falls) in the older adult. Clin Geriatr Med.

